# Distinct Shift in Beta-Cell Glutaredoxin 5 Expression Is Mediated by Hypoxia and Lipotoxicity Both *In Vivo* and *In Vitro*

**DOI:** 10.3389/fendo.2018.00084

**Published:** 2018-03-12

**Authors:** Sebastian Friedrich Petry, Lia Mingzhe Sun, Anna Knapp, Sabrina Reinl, Thomas Linn

**Affiliations:** ^1^Clinical Research Unit, Center of Internal Medicine, Justus Liebig University, Giessen, Germany

**Keywords:** diabetes mellitus type 2, glutaredoxin, islet remodeling, rodent diabesity, db mouse, MIN6, lipotoxicity, hypoxia

## Abstract

Histomorphological and functional alterations in pancreatic islet composition directly correlate with hyperglycemia severity. Progressive deterioration of metabolic control in subjects suffering from type 2 diabetes is predominantly caused by impaired beta-cell functionality. The glutaredoxin system is supposed to wield protective properties for beta-cells. Therefore, we sought to identify a correlation between the structural changes observed in diabetic pancreatic islets with altered glutaredoxin 5 expression, in order to determine an underlying mechanism of beta-cell impairment. Islets of db/db mice presenting with uncontrolled diabetes were assessed in terms of morphological structure and insulin, glucagon, and glutaredoxin 5 expression. MIN6 cell function and glutaredoxin 5 expression were analyzed after exposure to oleic acid and hypoxia. Islets of diabese mice were marked by typical remodeling and distinct reduction of, and shifts, in localization of glutaredoxin 5-positive cells. These islets featured decreased glutaredoxin 5 as well as insulin and glucagon content. In beta-cell culture, glutaredoxin 5 protein and mRNA expression were decreased by hypoxia and oleic acid but not by leptin treatment. Our study demonstrates that glutaredoxin 5 expression patterns are distinctively altered in islets of rodents presenting with uncontrolled diabesity. *In vitro*, reduction of islet-cell glutaredoxin 5 expression was mediated by hypoxia and oleic acid. Thus, glutaredoxin 5-deficiency in islets during diabetes may be caused by lipotoxicity and hypoxia.

## 1 Introduction

Type 2 diabetes mellitus is hallmarked by deprivation of the microarchitecture of pancreatic islets and progressive loss of beta-cells due to gluco- and lipotoxicity as well as a chronic state of inflammation (1). In particular, lipotoxicity, as mediated by free fatty acids, is a pivotal pathogenetic factor in type 2 diabetes as it induces pronounced insulin resistance (2) concomitant with significant impairment of insulin secretion (3). Free fatty acids mediate beta-cell death by induction of ER stress (4) and ROS production (5). During high metabolic activity, beta-cells further suffer from a hypoxia-like condition (6), which exhibits substantial damage to its secretory apparatus (7). However, the islets of Langerhans broadly express members of the glutaredoxin (Grx) system (8). These proteins are mainly involved in redox regulation of cellular processes and biogenesis of iron–sulfur proteins. Glutaredoxins (Grxs) are assumed to wield protective properties. Mammals express four Grxs classified as mono- or dithiol Grx dependent on the number of cysteine residues in their active center.

Dithiol oxidoreductases Grx1 and 2 are major actors in thiol-disulfide exchange (9–11). They exercise control over their targets by reversible posttranslational de-glutathionylation of cysteine residues in dependence of glutathione reductase and glutathione. Monothiol Grx3 and 5 have no known catalytic activity. They are essential for biogenesis of proteins containing iron–sulfur clusters (12, 13). Mitochondrial Grx5 is directly involved in the composition of iron–sulfur clusters and thereby essential for mitochondrial, as well as cytosolic iron–sulfur proteins, which are essential for cell function (14). Grx5-deficiency results in elevated susceptibility to oxidative and osmotic stress together with cellular iron overload in yeast (14, 15). In zebra fish lacking Grx5, these pathologies occur together with anemia (16). The relevance of Grx5 for unimpaired heme biosynthesis and iron homeostasis is also apparent in human erythroblasts. An example of the effects of Grx5-deficiency was demonstrated in a patient suffering from defective homozygous GLRX5 mRNA-splicing. This patient presented a distinct phenotype including diabetes mellitus mediated by pancreatic iron overload, indicating the crucial role of Grx5 for unimpaired glucose metabolism (17).

Rodents feature a defined composition of the islets of Langerhans. Predominantly, islets consist of insulin-producing beta- and glucagon-producing alpha-cells. The vast majority of beta-cell mass is located in islet center, while alpha-cells are located in islet periphery, together with delta-, gamma- and epsilon cells (18, 19). In humans, cells are distributed randomly throughout islets (20). It is well accepted that disruption of physiological islet cell compositions occurs in diabetes and has remarkable functional impact (21, 22). Apart from apoptosis, beta-cell loss involves dedifferentiation (23–25) and autophagocytosis, where by the islets undergo remodeling (26, 27). However, the exact mechanisms underlying islet remodeling are not entirely understood and apart from documented expression of Grxs in the islets of Langerhans there is little knowledge about their significance for islet physiology. Despite alpha-cell dysfunction in diabetes mellitus being well known in human subjects and rodents (28, 29), there are no publications addressing Grx expression in glucagon-producing cells. However, data indicate that in contrast to beta-cells, alpha-cells are well-provided with antioxidant enzymes (30).

The aim of this study was to determine whether; (I) islets of db/db mice presenting with uncontrolled diabetes differ qualitatively and quantitatively from lean, leptin-susceptible wild types in terms of Grx5 expression, (II) changes in islet Grx5 protein pattern correlate with structural alterations and shifts in the cellular composition of the islets of Langerhans, (III) leptin action can be delineated from changes in glutaredoxin expression *in vitro*, and (IV) hypoxia and lipotoxicity have an effect on beta-cell Grx5 expression.

We report reduced Grx5 content in islets, loss of insulin content, and loss of glucagon content in pancreases of db/db mice presenting with uncontrolled diabesity in comparison to lean, non-diabetic C57BL/6 littermates. Islet Grx5 patterns were associated with a reduction of structural complexity of islets. We also describe novel data for a connection between Grx5 and beta-cell insulin secretion capacity *in vitro*. Changes in beta-cell Grx5 content were shown to be independent from leptin-resistance but were dependent on hypoxia and oleic acid in a dose-dependent manner. This is the first report of distinct histomorphological alterations of islet Grx5 expression patterns during uncontrolled rodent diabesity in context with altered islet composition and a possibly lipotoxicity-mediated loss of beta-cell Grx5 expression.

## 2 Materials and Methods

### 2.1 Animal Model

15 male BKS(D)-Leprdb/JOrlRj (db/db) mice and 12 male C57BL/6NRj (C57) mice were acquired at the age of 10 weeks from Janvier Labs and were given 2 weeks to adapt to local animal facility. Number of required mice was calculated regarding manifestation rate of diabetes according to our previous study with type I error of 0.05 and type II error of 0.2. Housing conditions involved room temperature of 22 ± 0.5°C, 12 h light/dark cycle, 60% humidity, and tap water and standard diet pellet food (Altromin, Lage, Germany) *ad libitum* in individually ventilated cages in groups of five mice in accordance with institutional guidelines. Mice were sedated by isoflurane (5%). Thereafter, pancreas retrieval for histological studies was carried out at 12 or 13 weeks of age for db/db animals and 12 or 14 weeks of age for controls as diabetic animals were symptomatic and could thus not be kept for a prolonged period. Respective time points were pooled for both groups.

### 2.2 Histochemistry and Immunohistochemistry

Light microscopy was used for detection of insulin (Dako, Hamburg, Germany) and primary assessment of islet morphology. Organs were fixated with 3.5–3.7% formaldehyde, rinsed with 70% ethanol, and stored overnight. Embedding was carried out with paraffin after treatment in ascending alcohol series. Prior to staining, paraffin was removed using terpene (Roti-Histol, Roth, Karlsruhe, Germany) and descending alcohol series. Slides were washed with Tris and blocked with 1% goat serum for 20 min. Primary antibodies diluted in 1% goat serum dissolved in TBS containing 0.3% Triton X-100 (0.3% PBST) were applied overnight at 4°C. Secondary antibodies in 5% mouse serum were applied thereafter for 1 h at room temperature. Fuchsine red staining was used in order to visualize insulin. Staining progress was observed with light microscope and stopped after 1 min in Tris. Staining procedure was finished by counterstaining with hemalum–eosin 10% for 1 min (hemalum) and 5 min (eosin) before preservation and conservation with VectaMount (Vector Laboratories, Burlingame, CA, USA).

### 2.3 Immunofluorescence

Immunofluorescent staining was used for detection of insulin (Dako, Hamburg, Germany), Glucagon (Novus Biologicals, USA), and Grx5 (kindly provided and validated by Prof. Lillig/Dr. Hanschmann as described in Ref. (31)). Organs were stored overnight in PBS supplemented with 18% sucrose solution, embedded in cryoblock embedding medium (Biosystems, Nunningen, Switzerland) and frozen at −80°C. Organs were sectioned using Leica Crysostat CM1850 (Leica, Wetzlar, Germany) in order to acquire slides of 7 µm thickness. Frozen tissue was fixated with Zamboni (paraformaldehyde in picric acid and PBS as described in Ref. (32)) for 15 min. Slides were washed with Tris-buffer and blocked with 1% donkey serum dissolved in 0.3% TBST for 20 min. Incubation with primary antibodies diluted in 1% donkey serum dissolved in 0.3% PBST took place overnight at 4°C. Secondary antibodies in 5% mouse serum were applied for 1 h at room temperature. Nuclei were stained with Hoechst (Calbiochem, Darmstadt, Germany) in 0.1% TRIS buffer pH 7.6 and samples were preserved with ProLong Gold (Invitrogen, Karlsruhe, Germany).

Extracted pancreases were sectioned entirely. Manual optical assessment for quality was employed, i.e., slides with damaged structure were rejected. Multiple inclusion of the same islets was avoided by maintaining an interval of 140 µm between slides used for analysis and by manual comparison of islets. Appropriate comparability of immunohistological staining was achieved by preparation in batches. Slides were screened entirely, all islets were included. Successful staining of target antigen and avoidance of extensive background staining was verified by comparison against samples prepared without the respective primary antibody.

### 2.4 Measurement of Islet Area and Quantification of Fluorescent Signals

Images were taken with Leica Application Suite v 3.8.0 using digital microscope camera DFC 420 C (Leica, Wetzlar, Germany). Analysis of islet area and quantification of fluorescent signal of insulin, glucagon, and Grx5 was employed using custom scripts for ImageJ (Wayne Rasband, National Institutes of Health, USA) as described before (33). Briefly, ImageJ was calibrated to match image scale. Single islets were optically selected. Exact islet area was identified using combined overlay images (staining of nuclei, insulin, and glucagon). Islet region was carefully tagged manually by use of freehand selection according to outline of insulin and glucagon staining and typical clusters of stained nuclei. Area of selection was measured, and ROI of identified islets were saved for following analysis.

Absolute area of insulin, glucagon, and Grx5 staining per islet was acquired by applying a threshold value to previously saved ROI. Threshold value was manually adapted to limit selection to staining of the respective antibody. Area of selection was measured and ROI were saved for analysis of fluorescent intensity. Relative area of the respective target antigen per islet was normalized to respective islet area to achieve better comparability between islets of different area. As third parameter of staining area quantification, absolute Grx5 area was set in relation to absolute insulin area for every islet to correlate islet Grx with insulin content.

Quantification of fluorescent intensity of insulin, glucagon, and Grx5 as quantification of respective protein content was obtained by measuring mean fluorescent intensity in the previously identified ROI for areas stained by respective antibodies. Therefore, images were normalized by removing background using slides without primary antibodies and converted to gray scale. Fluorescent intensity was measured in emitted fluorescence and given in mean gray values ranging from 0 (0%) to 255 (100%) and normalized against islet extent to achieve better comparability between islets of different area.

Number of Grx5-positive cells per islet was quantified by manually counting all nuclei per islet and identifying those presenting Grx5 staining. Results were given in percentage of cells displaying Grx5 fluorescence.

For each analysis mean of three individual runs was calculated to limit influence of manual selection of ROI and adaption of threshold values.

### 2.5 Cell Culture and Protein Analysis

Mouse insulinoma cells 6th subclone (MIN6 cells) cell line was obtained from Dr. Sigurd Lenzen (Institute of Clinical Biochemistry, Hannover Medical School, Hannover, Germany) (34) (originally from Dr. Miyazaki, Institute for Medical Genetics, Kumamoto University Medical School, Japan (35)) and cells were routinely maintained in Dulbecco’s modified Eagle medium (DMEM, Life Technologies, Darmstadt, Germany) containing 25 mM glucose, supplemented with 10% fetal calf serum (biowest, Nuaillé, France), 2 mM l-glutamine, 25 mM Hepes (Biochrom, Berlin, Germany), 285 µM 2-mercaptoethanol (Life Technologies, Darmstadt, Germany), and 1% penicillin/streptomycin (Life Technologies, Darmstadt, Germany). Subculture and maintenance were performed as reported repeatedly in publications from our group (36, 37). MIN6 cells presented in this study were at passages 50-60. We compared earlier passage cells (P < 30) and they did not differ in normal glucose stimulated insulin secretion (GSIS) from P > 30 (data not shown). All assays used MIN6 cells grown to 70-80% confluence unless otherwise stated.

Cells were cultured at 37°C and 5% *CO*_2_ and split by trypsinization. Washing was done with PBS before adding 0.5% Trypsin-EDTA (Gibco, Darmstadt, Germany) solution. Detachment was carried out by dilution with DMEM and centrifugation for 4 min at 1,200 RPM before seeding into new flasks.

For leptin cultivation, prior to analysis recombinant mouse leptin (R&D, Wiesbaden, Germany) was applied for 2 and 48 h, respectively. Leptin was pre-diluted to 0.1% in 20 mM Tris–HCl, pH 8.0. Concentrations of 0, 0.075, 0.45, and 2 ng/ml were applied. For fatty acid treatment, oleic acid was applied for 24 h under normoxic and hypoxic (2% *O*_2_) atmosphere, respectively. Concentrations of 0, 0.5, and 0.75 mM were applied, respectively. Lysates and supernatant was collected for ELISA/PCR analysis.

Protein expression for insulin and Grx5 was measured in MIN6 cell lysates and supernatant. Prior to lysis, 1 ml supernatant was extracted before cells were washed in ice-cold PBS. Cells were incubated on ice for 20 min in NP-40 lysis buffer (United States Biological, Swampscott, USA). Supernatant was gathered by centrifugation for 20 min at 1,200 RPM. Insulin (DRG Instruments GmbH, Marburg, Germany) and Grx5 (CUSABIO Biotech, Wuhan, China) content was analyzed using ELISA technique. As Grx5 concentration exceeded largest measured standard (1,609.05 pg/ml) at 48 h leptin treatment, data were extrapolated. As absorbance in ELISA reached a plateau, it has to be noted that Grx5 protein level at 2 ng/ml leptin treatment was omitted for analysis.

Cell viability was assessed by Vybrant MTT Cell Proliferation Assay Kit following manufacturer’s instructions (Molecular Probes, Inc., Waltham, MA, USA).

### 2.6 RNA Isolation, cDNA Synthesis, and qRT-PCR

MIN6 cell RNA was extracted using RNeasy Plus Micro Kit (Qiagen, Düsseldorf, Germany). Total RNA concentration was determined by OD260 nm method using NanoDrop 1000 spectrophotometer (Thermo Scientific, Schwerte, Germany). SuperScript III Reverse Transcriptase kit (Invitrogen, Darmstadt, Germany) was employed to synthesize cDNA. qRT-PR was carried out on real-time PCR System StepOnePlus (Applied Biosystems). PCR included 615 s of activation/denaturation at 95°C and 40 cycles of annealing and elongation (95°C, 30 s each). Primer (Invitrogen, Darmstadt, Germany) concentration for qRT-PCR was 20 pM. Sequences were as follows:

beta-actin (housekeeping in leptin culture):
fwd CGT GAA AAG ATG ACC CAG ATC A, rev CAC AGC CTG GAT GGC TAC GT;
rpl32 (housekeeping in oleic acid culture):
fwd GGA GAA GGT TCA AGG GCC AG, rev GCG TTG GGA TTG GTG ACT CT;
Grx5:
fwd GAA GAA GGA CAA GGT GGT GGT CTT C, rev GCA TCT GCA GAA GAA TGT CAC AGC

Relative mRNA expression values were obtained by normalizing CT values of the target genes in comparison with CT values of the housekeeping gene using the delta-CT method.

### 2.7 Statistical Analysis

Statistical analysis was performed using Graph Pad Prism 5 (GraphPad Software, San Diego, CA, USA) using Mann–Whitney test and two-way ANOVA as appropriate. Data are given as mean values ± SEM, with n denoting the number of experiments unless otherwise indicated. A p-value < 0.05 was considered significant.

## 3 Results

### 3.1 Uncontrolled Diabesity on the Genetic Background of the db Mutation

**Figure 1:**
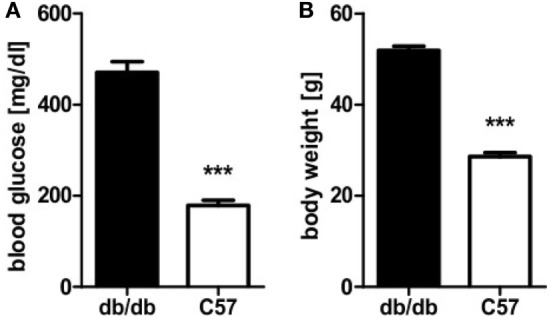
Blood glucose level and body weight of db/db and C57BL/6 mice. **(A)** Blood glucose level of db/db and C57BL/6 mice. **(B)** Body weight of db/db and C57BL/6 mice. Values are mean ± SEM (n = 12–15 mice), black bars represent db/db mice, white bars represent C57BL/6 mice, *** denotes *p* < 0.0001.

Obese homozygous leptin-resistant db/db mice were utilized for comparison with lean wild-type C57BL/6 animals as the db strain is typically presenting a strong phenotype of diabesity. Homozygotes featured uncontrolled diabetes mellitus with polyuria and weight loss exhibiting significantly (*p* < 0.0001) higher blood glucose levels than their lean, non-diabetic littermates. Mean blood glucose value was 471 mg/dl and thus 2.6-fold higher than in controls whose average blood glucose level was 179 mg/dl (Figure 1A). Furthermore, a distinct phenotype of rodent obesity was still apparent despite beginning weight loss in db animals. Average body weight was 52 g in db/db animals in comparison to 29 g in controls (*p* < 0.0001, Figure 1B).

### 3.2 Loss of Islet Structure and Elevated Islet Area in Rodent Diabesity

**Figure 2:**
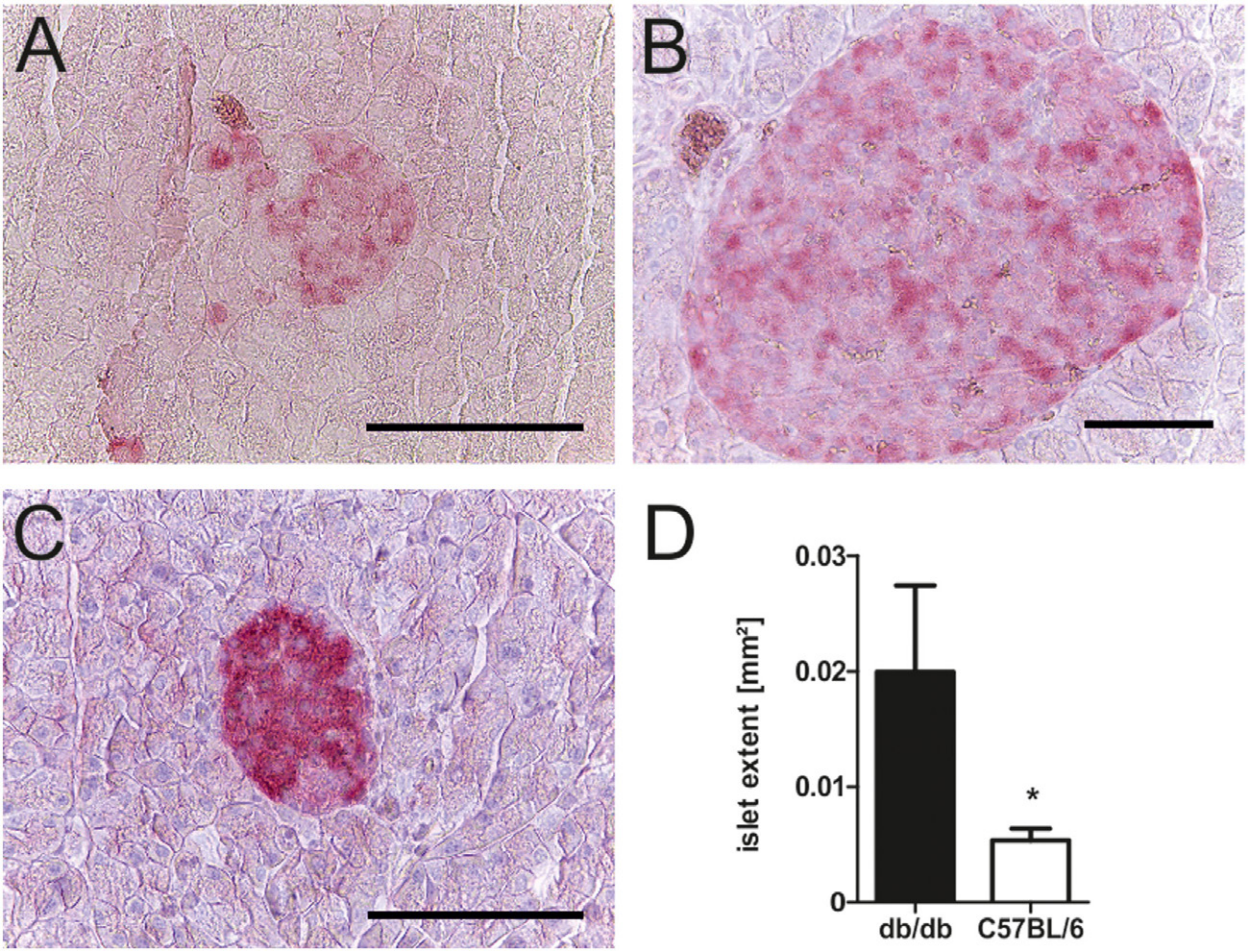
Morphology and quantification of db/db and C57BL/6 islet extent. **(A–C)** Representative images taken of db/db and C57BL/6 islets using light microscopy in comparison: **(A)** typical small db islet with lost demarcation to exocrine issue, **(B)** example of extensive db islets, and **(C)** generic C57 islet (fuchsine red: insulin, bars indicate 50 µm). **(D)** Quantification of mean islet area as measured with ImageJ corresponding to islet extent. Black bars represent db/db mice, white bars represent C57BL/6 mice, n = 6 mice, * denotes *p* < 0.05.

To relate the phenotype of uncontrolled diabetes to histomorphological alterations, islet structure was assessed histologically. Light microscopy was used as it allowed for easier discrimination between endocrine and exocrine tissue. Analysis revealed marked differences between islets of diabetic db/db mice and non-diabetic C57BL/6 controls. Pancreases of db animals contained two types of islets: small deformed ones with lost demarcation to exocrine tissue (Figure 2A) as well as a vast number of remarkably extensive islets (Figure 2B). In contrast, C57 islets were clearly defined from exocrine tissue and presented a notably lower variability of islet size (Figure 2C). The optical impression of elevated islet extent was confirmed by quantification of islet area using stained slides. Despite high variability, islets of db/db mice were significantly larger (*p* < 0.05) than C57 islets (Figure 2D), on average by 1.5-fold.

### 3.3 Shift in Islet Cell Composition As a Sign of Uncontrolled Diabetes

**Figure 3:**
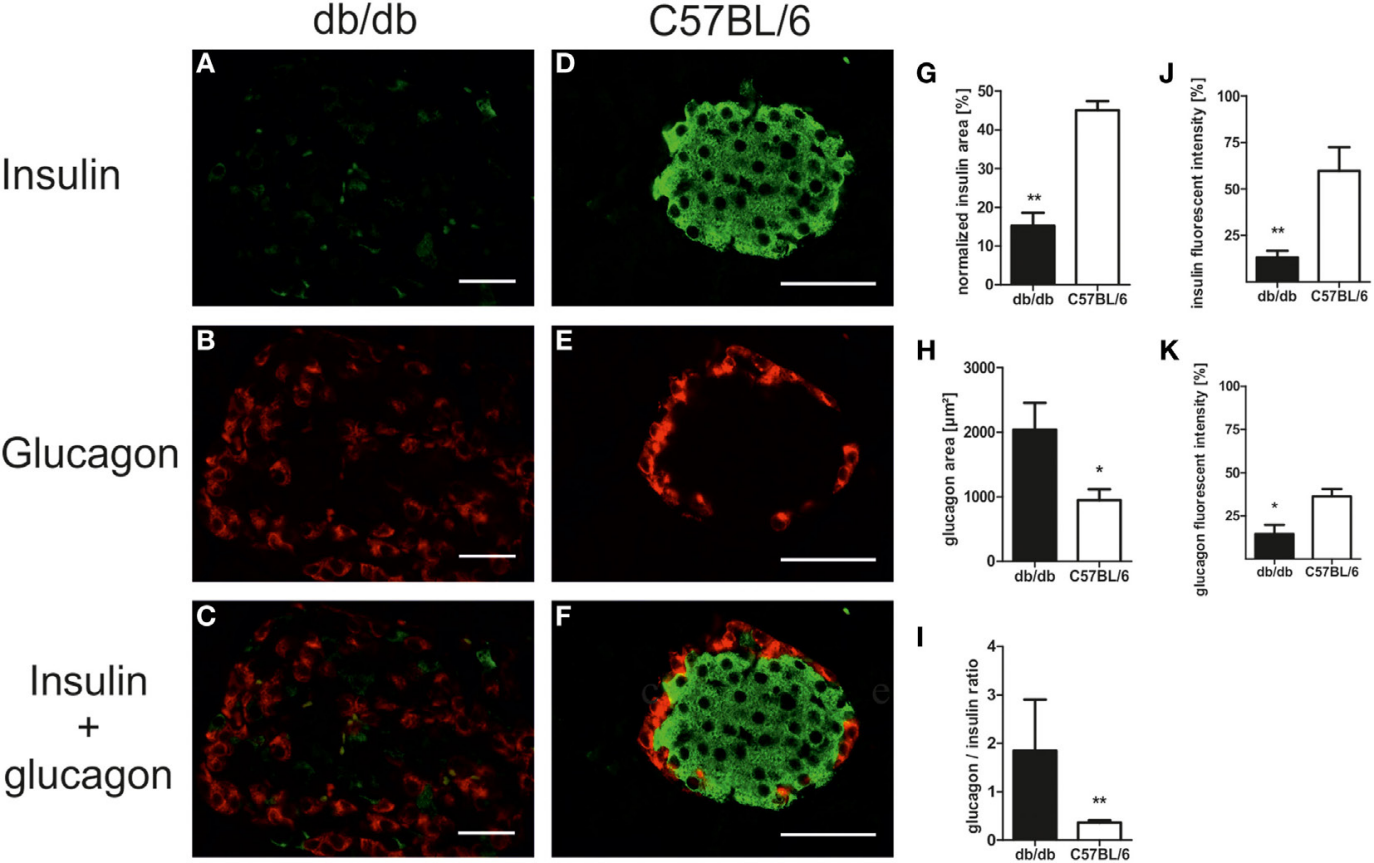
Islet composition and insulin/glucagon content of db/db and C57BL/6 islets. **(A–F)** Representative images taken of immunostained db/db and C57BL/6 islets stained for insulin and glucagon (green: insulin, red: glucagon, bars indicate 50 µm). **(G,H)** Quantification of relative islet insulin and absolute glucagon area. **(I)** Quantification of islet glucagon/insulin area ratio. **(J,K)** Quantification of insulin and glucagon fluorescent intensity. Black bars represent db/db mice, white bars represent C57BL/6 mice, n = 6 mice, ** denotes *p* < 0.005, * denotes *p* < 0.05.

In line with the diabetic phenotype images acquired for morphological studies also revealed faint insulin appearance in db/db when compared to the intense staining of C57BL/6 islets (Figures 3A–F). To further evaluate cell-specific pathologies, immunofluorescence staining of insulin and glucagon was conducted. This allowed for individual assessment of islet beta- and alpha-cell areas per islet and estimation of islet insulin and glucagon content from the same section.

Analysis of immunostained slides indicated a shift in islet cell composition in db/db animals. Islets featured staining of alpha-cells in both the periphery and center with an overall increase in alpha-cell area as defined by positive staining of glucagon (Figures 3A–C). By contrast, C57 islets presented typical murine distribution patterns of peripheral alpha-cells (Figures 3D–F). Quantification of insulin and glucagon area per islet exposed a non-significant trend for higher absolute insulin area in non-diabetic wild-type mice (data not shown). However, relative insulin area normalized to islet extent demonstrated a significantly (*p* < 0.005) decreased value in db/db islets (Figure 3G). In contrast, absolute glucagon area in diabetic animals was significantly (*p* < 0.05) elevated (Figure 3H), while normalized glucagon area showed no significant difference between both strains of mice (data not shown). This shift in alpha- to beta-cell mass ratio in islets of diabetic mice was significant despite high variability in db/db islets (*p* < 0.005, Figure 3I).

Apart from this alteration of islet alpha- and beta-cell area, loss of staining intensity for insulin as well as glucagon was apparent. Quantitative analysis of fluorescent intensity was employed to assess islet insulin and glucagon content, confirming the optical impression of fainter staining in db/db islets with significantly lower fluorescent intensity for both proteins (*p* < 0.005 for insulin, *p* < 0.05 for glucagon, Figures 3J,K).

### 3.4 Distinct Grx5 Expression Patterns in Diabesity

In order to correlate the observed phenotypical and morphological differences between leptin-resistant, diabetic db/db animals and leptin-susceptible, non-diabetic controls with changes in expression of Grx5, we assessed islet Grx5 staining patterns in both strains of mice.

**Figure 4:**
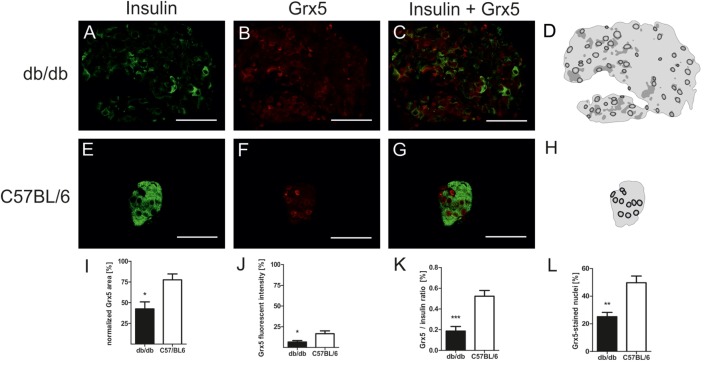
Grx5 expression patterns in db/db and C57BL/6 mice and quantification of Grx5 area, fluorescent intensity, Grx5 to insulin ratio and nuclei staining. **(A–C,E–G)** Representative images taken of immunostained db/db and C57BL/6 islets stained for insulin and Grx5 (green: insulin, red: Grx5, bars indicate 50 µm). **(D,H)** Schematics of Grx5 staining in db/db and C57BL/6 islets (black: high intensity staining to white: no signal detected). **(I)** Quantification of islet Grx5 area normalized to islet extent. **(J)** Quantification of Grx5 fluorescent intensity. **(K)** Quantification of Grx5 to insulin ratio. **(L)** Quantification of nuclei stained for Grx5. Black bars represent db/db mice, white bars represent C57BL/6 mice, n = 6 mice, *** denotes *p* < 0.0001, ** denotes *p* < 0.005, * denotes *p* < 0.05.

Both strains of mice featured global staining of islets cells with emphasis on nuclei as identified by comparing Grx5 and nucleus staining (Figures 4A–H). However, in db/db islets detection of Grx5-positive cells with nuclear staining was not as striking as in C57BL/6 islets. They exhibited a rather global and indistinct distribution of Grx5 with decreased intensity, and significantly (*p* < 0.005) lower Grx5-positive cell count. Quantification resulted in, on average, 25% Grx5-positive cells with nuclear staining in db/db islets and 49% in C57 islets (Figure 4L). In correspondence to generally enlarged islet size, diabetic mice were marked by significantly (*p* < 0.05) elevated Grx5 area (data not shown). When Grx5-positive cell area was normalized to islet extent this relation was reversed and a significant (*p* < 0.05) relative loss of Grx5 immunostaining was observed (Figure 4I). The differences in Grx5 expression between diabese and non-diabetic, lean controls could be further defined by quantification of Grx5 fluorescent intensity. Fluorescent intensity was significantly reduced in db/db islets, despite an increase in the area of positively stained cells, correlating with reduced islet Grx5 content (Figure 4J). The Grx5 to insulin content ratio was significantly (*p* < 0.0001) reduced in db/db islets (Figure 4K).

### 3.5 No Effect of Leptin on Grx5 Expression in Mouse Beta-Cells

**Figure 5:**
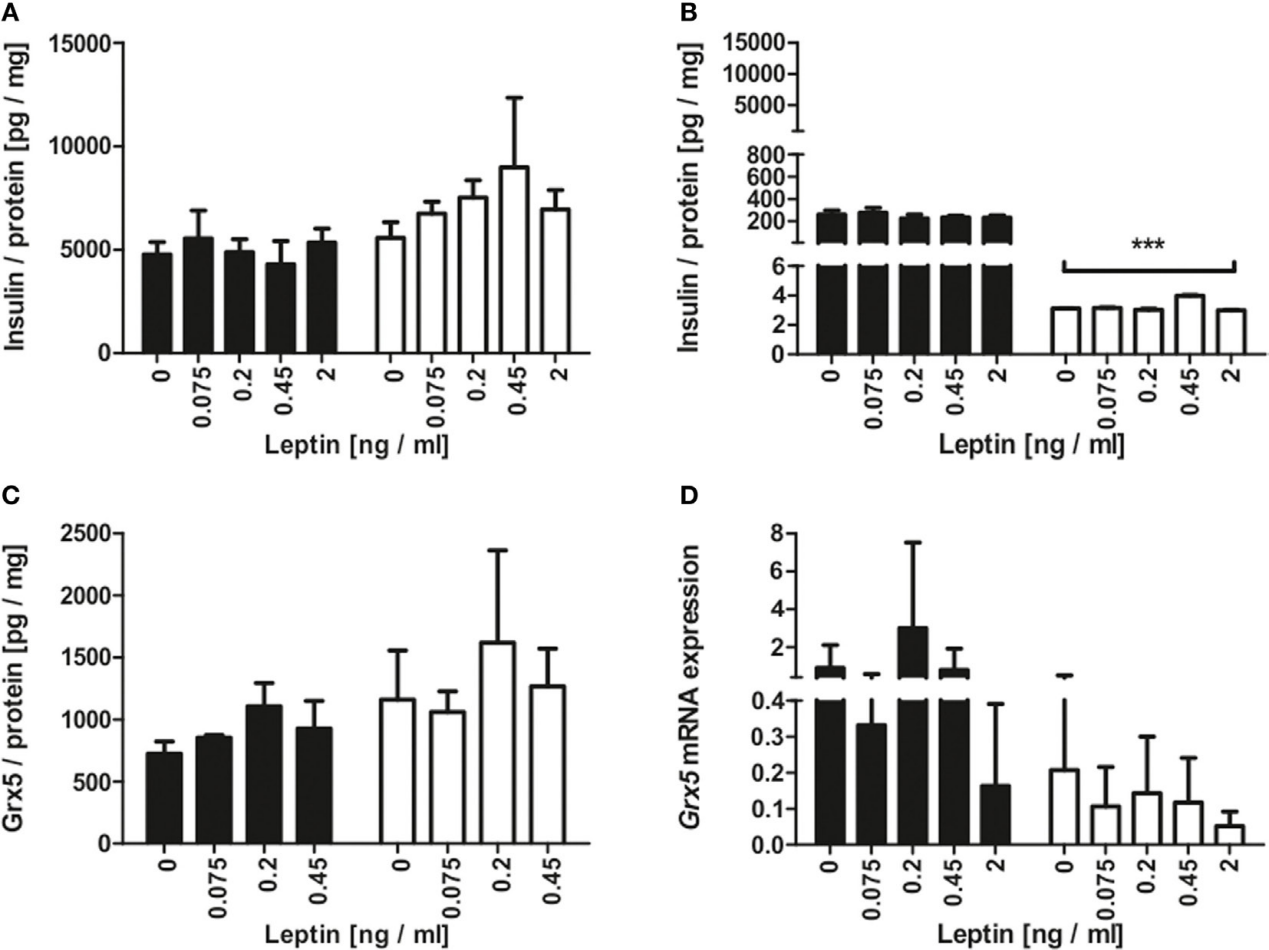
Insulin and Grx5 protein expression and *Grx5* mRNA expression in MIN6 cells upon exposure to recombinant leptin for 2 and 48 h. **(A)** MIN6 lysate insulin content. **(B)** MIN6 supernatant insulin content. **(C)** MIN6 lysate Grx5 content. **(D)** MIN6 *Grx5* mRNA expression. Black bars represent 2 h, white bars represent 48 h. n = 9 runs, *** denotes *p* < 0.001.

Since the homozygous db mouse is an extreme example of total leptin resistance, our aim was to study whether leptin exposure to mouse beta-cells has influence on Grx expression. MIN6 cells were incubated with several concentrations of recombinant leptin for 2 and 48 h. As a control, MIN6 insulin content (Figure 5A) was measured in cell lysates and insulin secretion measured (Figure 5B) in supernatant. While insulin secretion declined significantly after 48 h of culture, no effect of leptin on insulin secretion was detected. Grx5 protein and mRNA expression were studied in MIN6 cells exposed to leptin. Grx5 protein expression featured a time-dependent non-significant increase toward 48 h which was independent of leptin concentration (Figure 5C). *Grx5* mRNA expression decreased with time and was also not influenced by leptin (Figure 5D). Grx5 was not detected in cell supernatant (data not shown).

### 3.6 Beta-Cell Grx5 Expression Is Regulated by Hypoxia and Oleic Acid

**Figure 6:**
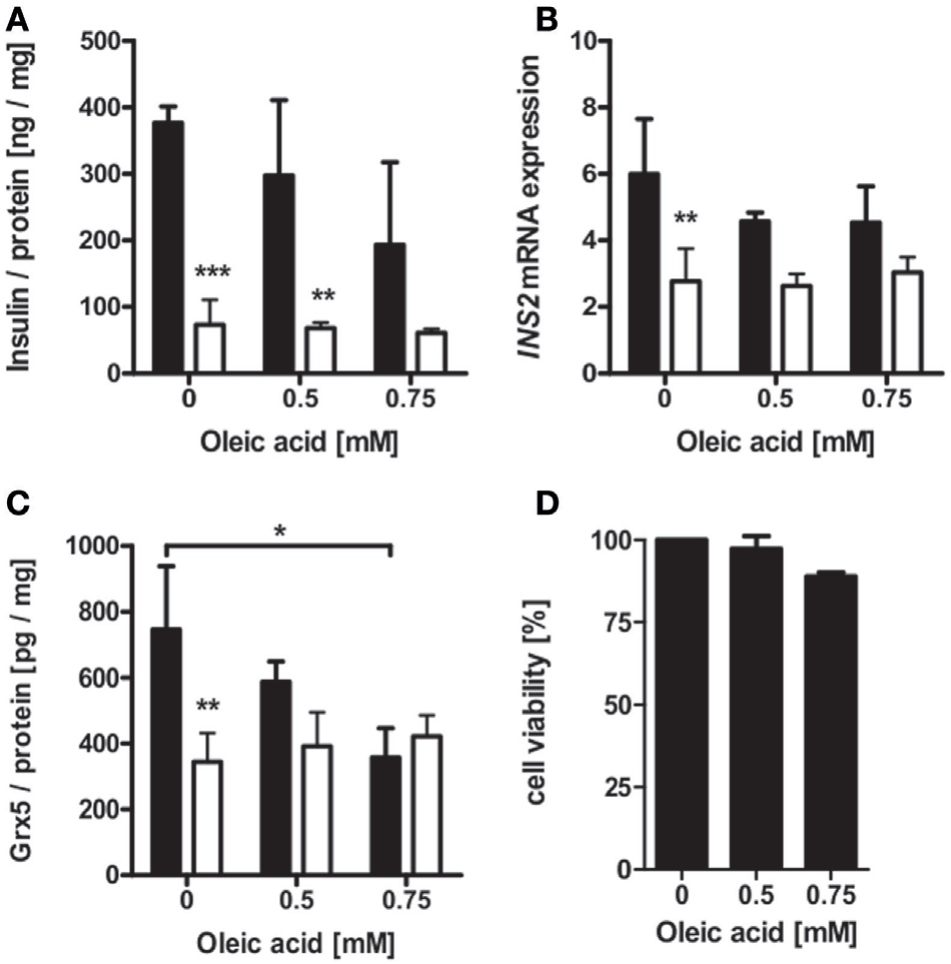
Insulin and Grx5 protein and mRNA expression in MIN6 cells upon exposure to hypoxia (2% *O*_2_) and oleic acid for 24 h. **(A)** MIN6 supernatant insulin content. **(B)** MIN6 *INS2* mRNA expression. **(C)** MIN6 lysate Grx5 content. **(D)** MIN6 MTT assay after 24 h of normoxic oleic acid treatment. Black bars represent normoxic atmosphere, white bars hypoxic atmosphere. n = minimum of 3 runs. *** denotes *p* < 0.001, ** denotes *p* < 0.01, * denotes *p* < 0.05.

Islet isolation of diabetic db/db mice presenting with uncontrolled diabesity for protein and mRNA analysis was not successful, due to the low yield of intact islets from db/db pancreases. Possible mechanisms behind deficiency of islet Grx5 content were, therefore, evaluated *in vitro*. MIN6 cells were exposed to oleic acid and hypoxia (2% *O*_2_) for 24 h. MIN6 secretion as measured by insulin content in cell supernatant was significantly attenuated under hypoxic treatment (*p* < 0.001). Exposure to oleic acid resulted in a non-significant trend of reduced insulin secretion (Figure 6A). Hypoxia significantly (*p* < 0.01) reduced *INS2* mRNA expression, whereas treatment with oleic acid resulted in decreased expression; however, this was not significant (Figure 6B). Functional impairment of MIN6 cells upon hypoxic and oleic acid treatment was then correlated with Grx5 protein content and mRNA expression. In concordance with reduced insulin secretion and mRNA expression, MIN6 cells presented significantly reduced Grx5 protein content upon hypoxic (*p* < 0.01) and oleic acid (*p* < 0.05) incubation (Figure 6C). Both treatments attenuated *Grx5* mRNA expression; however, effects were not significant (data not shown). To exclude beta-cell decay as a mediator for secretory impairment and reduction of Grx5, an MTT assay was conducted. MIN6 cells maintained strong viability upon exposure to oleic acid for 24 h under normoxia (Figure 6D).

## 4 Discussion

The glutaredoxin system has been repeatedly reported to exert protective effects on pancreatic beta-cells. In this study, we aimed to further elucidate these findings and evaluate a possible connection to histomorphological deviations of the islets of Langerhans occurring during uncontrolled diabesity as well as possible underlying mechanisms. Diabetic mice suffering from leptin-resistance on the db/db background were employed as a rodent model for diabetes mellitus type 2 and C57BL/6 wild-type animals without impairment of leptin signaling served as controls. As the utilized strain of db/db mice originates from a mixture of the C57BL/KsJ substrain and C57BL/6 wild-type mice (38, 39), they share a genetic background and thus provide a reasonable mouse model for our experimental setting.

A shift in islet cell composition associated with experimental diabetes is well documented (21, 22, 40, 41), but the underlying mechanisms are not fully understood. However, there is evidence that altered beta-cell functionality is profoundly involved in these histomorphological changes of the insulin-producing pancreatic micro organs. For instance, beta-cells were shown to express markers typically associated with endocrine progenitor cells in diabetes. Marked beta-cell plasticity was observed during progression of hyperglycemia as researchers reported beta-cell dedifferentiation into alpha-cells (23–25). Accordingly, beta-cells of hyperglycemic rodents including db/db mice feature loss of transcription factor Forkhead box protein O1 (FoxO1), an important regulator of beta-cell mass and stress response, which is accompanied by dedifferentiation into alpha-cells (23). Furthermore, levels of oxidative stress rise in pancreatic islets during diabetes (42). Oxidative stress is an acknowledged trigger of islet dysfunction (43) and maintenance of beta-cell differentiation is challenged by oxidative conditions (44). Islets of db/db mice typically present altered antioxidant enzyme expression, apart from Grxs (45), and modulation of oxidative stress markers (46) as well as increased ROS (33). Hypoxia has been described to occur in the beta-cells during high metabolic activity due to immensely high levels of oxidative phosphorylation (6, 7). As a result, a HIF-mediated hypoxic stress response is triggered (6) and apoptosis (6), upregulation of NF-κB, and oxidative stress (36) occur. Beta-cells are exceptionally vulnerable to hypoxia. In consequence, db/db islets present a marked increase in HIF and HIF target gene transcription (47) as well as NF-κB-mediated apoptosis (37). Free fatty acids have been reported to interfere significantly with beta-cell turnover (48) and induce functional and structural aberration of islets (49). These data indicate that the observed shift in islet cell composition is directly linked to impaired islet functionality mediated by gluco-/lipotoxicity and hypoxia. As a result, islets of db/db mice present pronounced structural deviations marked by a considerable amount of degranulated, i.e., endocrine inactive, beta-cells (50). Interestingly, Grxs are involved in regulation of oxidative stress, HIF (51) as well as NF-κB (52, 53). Beneficial effects of the Grx system on insulin secretion (54–56), as well as its impact on key enzymes of glucose metabolism (57–59), have been described. However, their role in pancreatic beta-cell function remains vague.

In the present histomorphological study, we identified distinct staining patterns of Grx5 with notable differences in expression between diabese mice and lean controls. Main features of Grx5 staining involved rarefication and loss of staining area as well as fluorescent intensity and Grx5 to insulin ratio in islets of diabetic mice. Strikingly, Grx5 presented a distinct staining of nuclei, which was mostly lost in diabetic islets. While Grx5 is an acknowledged mitochondrial glutaredoxin in yeast (14), it has been detected in rodent nuclei (8). However, the significance of this finding has yet to be elucidated. Grx5 is so far not known to exhibit catalytical activity, but Grx5-deficiency increases the susceptibility to oxidative stress (15) and correlates with cellular iron overload (17). These findings might account for Grx5 loss upon extensive metabolic stress; however, the observed loss of nucleic localization requires further study.

The homozygous db mice used in this study are hallmarked by complete leptin resistance (60) and thus exhibit a severe phenotype of diabesity (61). Adipokines and adipose-derived hormones are acknowledged mediators of inflammation, glucose and lipid metabolism, and energy balance (62). Leptin is an extensively studied hormone deriving from white adipose tissue (63). It is a key regulator of energy homeostasis (64) and exerts regulatory control on glucose metabolism and on the beta-cell itself in the so-called adipo-insular feedback loop. Leptin inhibits insulin secretion (65) and gene expression (66). In contrast, insulin increases leptin secretion (67) and gene expression (68). Expression of leptin correlates with fat mass leading to elevated levels in obese subjects (69, 70) as well as rodents (71). Homozygous db mice present complete leptin resistance. Therefore, influence of leptin excess on db/db islet Grx expression is highly unlikely. Our experimental setting was unable to deduce effects of leptin on insulin secretion or on Grx5 expression. In rodents, leptin plasma levels ranging from below 0.5 (72) up to 4,000 ng/ml (73) have been described. We deliberately chose leptin concentrations in a low physiological range to assess physiological influence on Grx5. Significant alterations of MIN6 insulin content and secretion occurred over time, as expected. While this poses a contradiction toward the adipo-insular axis, there are several differing reports in the current body of literature. Highly contingent on experimental settings and leptin dosage, studies reported no stimulation (74), stimulatory (75), and dose-dependent U-shaped responses in beta-cell lines, perfused pancreases, and isolated islets (76–78).

Interestingly, beta-cell Grx5 protein level correlated with insulin content. A non-significant decrease in *Grx5* mRNA occurred over time, which we interpreted as a sign of sufficient Grx5 protein levels in beta-cells under physiological conditions in contrast to fading expression in islets of diabese animals. To elucidate a possible mechanism behind reduced Grx5 levels, MIN6 cells were exposed to hypoxia and oleic acid. Oleic acid as a monounsaturated fatty acid is typically elevated in the diabetic metabolism of db/db mice (79) and is a mediator of lipotoxicity. Concentrations characteristically occurring in diabetes (2, 80, 81) were used in beta-cell culture. Both stressors induced significantly attenuated Grx5 protein levels in MIN6 cells in correlation with reduced insulin secretion *in vitro*. Thus, Grx5 might be depleted in diabese rodents as part of the beta-cell’s extensive stress response to lipotoxicity and hypoxia. Especially as a mitochondrial protein, beta-cell and islet Grx5 content might further be reduced due to structural and functional deviations of mitochondria typically occurring in type 2 diabetes (82). Elevated mitochondrial ROS caused by lipotoxicity and hypoxia might be the link between mitochondrial damage and decreased levels of Grx5. Increased susceptibility to oxidative damage and impaired iron-homeostasis with iron-mediated ROS catalyzation as caused by Grx5-deficiency (15, 17) would even fuel this process.

Given our present data and little available literature, we can only speculate as to a connection between pronounced adjustment in Grx expression and islet remodeling under metabolic stress. Further studies are required, particularly functional *in vivo* experiments to gain deeper insights into the specific role of Grx5 for the islets of Langerhans. Glutaredoxin-regulated pathways involved in beta-cell physiology as well as targets and effectors of Grx need to be identified. No causal link between islet Grx expression and islet remodeling has been previously reported, but we have shown that it represents a promising target for future research. However, we were able to demonstrate clear qualitative and quantitative deviations in histomorphological glutaredoxin patterns in uncontrolled rodent diabesity when compared to lean wild-type controls and we were able to delineate the phenomenon from leptin resistance *in vitro*. Oleic acid and hypoxia were identified as possible mediators of beta-cell and islet Grx5-deficiency. The influence of further typical stressors present during diabesity on beta-cell Grx5 expression should be evaluated using a broader range of dosage. Results could hereafter be validated using a different rodent model. In addition, the direct link between hypoxic/lipotoxic stress and reduced islet Grx5 must be evaluated *in vivo*. The potential influence of HIF-dependent cell remodeling and gene transcription as source of altered endocrine beta-cell function should be ruled out.

In conclusion, we provide evidence for a connection between Grx5 expression and islet dysfunction during diabesity *in vivo* as well as a direct link between Grx5 and beta-cell insulin secretion which is independent from leptin resistance. *In vitro* culture of beta-cells under the influence of hypoxia and oleic acid reveals a potential mechanism of action. Our data support the role of Grx5 as a protective factor for the pancreatic beta-cell.

## Ethics Statement

This study was carried out in accordance with the recommendations of institutional animal welfare officer, Chair of Animal Welfare of the Justus Liebig University Giessen, and Regional Administrative Council Giessen, Veterinary Department, under the code GI20/11-Nr.A18/2010. Both committees approved the protocol. All experiments were performed in accordance with German Animal Welfare Law. 3Rs were applied for reducing the number of required mice and reduce potential suffering, enrichment was applied to the IVC. Scoring for adverse events was done daily. Mice were anesthetized with isoflurane using a desiccator and sacrificed by cervical dislocation before removing the pancreas and other organs.

## Author Contributions

Conceptualization: SFP and TL. Data curation, formal analysis, investigation: SFP, LMS, AK, and SR. Writing—original draft preparation: SP. Writing—review and editing: SFP, LMS, AK, SR, and TL. Guarantor of the research: TL.

## Conflict of Interest Statement

The authors declare that the research was conducted in the absence of any commercial or financial relationships that could be construed as a potential conflict of interest.
